# How Much Iranian Parents Know of and How Well They Practice Regarding Antibiotics?

**DOI:** 10.22037/ijpr.2020.113695.14433

**Published:** 2021

**Authors:** Zahra Sharif, Fateme Babaei, Nazila Yousefi, Yasaman Alavian, Farzad Peiravian

**Affiliations:** a *Department of Pharmacoeconomics and Pharma Management, Faculty of Pharmacy, Shahid Beheshti University of Medical Sciences, Tehran, Iran. *; b *Faculty of Pharmacy, Shahid Beheshti University of Medical Science, Tehran, Iran.*

**Keywords:** Antibiotics, Parents, Knowledge, Practice, Iran

## Abstract

Irrational use of antibiotics as a global health concern has led to excessive treatment costs, failure of treatments, and antimicrobial resistance. Parents’ knowledge of and practice regarding antibiotics are two important factors contributing to the (ir) rational use of antibiotics. This study aimed to evaluate ill children’s parents’ knowledge and practice of prescribed antibiotics. Our subjects included only parents with children up to 12 (inclusive) years of age in Tehran, Iran’s capital. A prospective cross-sectional survey was conducted at 203 health care centers in Tehran. Parents’ knowledge was evaluated using a 37-item researcher-made questionnaire, and their practice about antibiotics was measured with a self-stated questionnaire, followed by an observational method to gain a real insight into their practice. SPSS 22 was used to analyze the data. A total of 401 randomly selected parents were enrolled in the study. The average score of parents’ knowledge of antibiotics regarding administration, indications, storage, and antimicrobial resistance was found to be 9.72 out of 17.00. In the self-stated method, an appropriate practice of antibiotic use was reported in 49.4% of participants who also got an average score of 3.95 points out of a total 8. In the observational method, most parents’ practice (68.4%) regarding antibiotic use was found to be acceptable; their average score was 5.93 points out of a total of 10. The findings of this study showed that half of the parents had adopted an acceptable practice regarding antibiotic use.

## Introduction

After the discovery of antibiotics, the mortality rate of infectious diseases (malaria, brucellosis, typhoid, *etc.*) sharply decreased (1). However, antimicrobial resistance (AMR) posed a new threat to our health, which was no less grave than that of the diseases themselves. People’s habits and practices regarding antibiotic use are also of great importance as not only does AMR occur naturally over time, but also people’s performance, attitude, and knowledge in (ir)rational use of antibiotics can be a very important contributing factor (2).

AMR is a global and serious health concern and an issue of high priority for WHO (World Health Organization) and healthcare systems (3, 4). Against this backdrop, the importance of the rational use of medicines, especially antibiotics, cannot be over-emphasized. Irrational use leads to excessive treatment costs, failure of treatments, and increased mortality and hospitalization rates (5).

Nowadays the borders of health demand have expanded, and the main aim of health policies is maintaining, expanding, and upgrading human health in societies, which can go a long way toward establishing social justice (6). Besides, people’s role is emphasized as the main factor contributing to self-health management; therefore, people must be aware of their roles, participate actively and wisely in their health and treatment decisions. One way for improving the rational use of medicine is enhancing patients’ health literacy, their ability to read and understand medication orders, and raising awareness of the rational use of antibiotics and AMR (7). 

Patients’ misunderstanding or poor comprehension of medication orders written on medicines – including daily dosage of the drug, time of use, number of medicines to be taken, medicines to be taken on an empty or full stomach, or mode of administration (to be chewed, swallowed, *etc*.) –, can be the main causes of medication errors, AMR, and treatment failures (7). Therefore, improvement in medication use and treatment is highly related to ill children’s parents’ raised general knowledge about the rational use of prescribed medicines (8, 9). Many pediatric providers have pointed out parents’ insistence on prescribing antibiotics for their children, though they might not need them at all (10). Misconceptions about antibiotics and treatment of respiratory diseases are common among parents as well (11). This issue becomes so crucial that children are the largest group of patients who receive antibiotics (12).

Iran is also facing bacterial resistance due to the irrational use of antibiotics (13), and despite many considerable measures taken in recent years, there are still many problems regarding the rational use of antibiotics (14). Therefore, we conducted a survey to assess parent’s literacy and practice regarding the rational use of antibiotics for their children to gain a deeper insight into the situation and identify educational gaps. 

## Experimental

*Method*

This study was a prospective cross-sectional questionnaire-based survey. Inclusion criteria were the basic literacy of reading and writing, having a child under 12 years old, referring to health centers, and living in Tehran. Exclusion criteria were unwillingness to corporate in the survey or failure to fully address all the items of the questionnaire. Participants also openly expressed their consent to take part in the survey and were assured their answers would be treated strictly confidential. Health centers in this study consisted of public and private hospitals and private physician’s offices. Out of 384 health centers in Tehran, based on Cochrane rule we had to pick up at least 200 centers. We assumed that some centers might not cooperate with researchers, therefore to assure that we would have that amount of centers at the end of the survey, 203 centers were randomly selected in different distinct of the city according to cluster sampling. Two parents from each selected centers entered the study, from which 401 completed the survey.

Parents’ knowledge and practice about antibiotics were measured via a 37-item researcher-made questionnaire. The reliability and validity of the questionnaire had been proven before the study started. The content validity index of the questions was greater than 0.79; therefore, according to Waltz and Basel method, they were valid. As for content reliability, we used Cronbach alpha, which accounted for 0.87 (15).

The questionnaire was composed of three parts. Part one consisted of the demographic characteristics of the respondents (age, sex, occupation, *etc*.) (Table 1). Part two assessed parents’ knowledge about antibiotics via 19 questions (Table 2). Moreover, the last part measured parents’ antibiotic practice, employing two methods: 1) a self-stated questionnaire which set out to evaluate parents’ perception of prescriptions and drug orders (8 questions) and 2) an observational method which set out to assess parents’ actual practice and how well they prepared antibiotics. Finally, data analyses were performed using the SPSS 22 software.

## Results

The number of participants who completed the whole survey, was whittled down to 401 most of whom were women with a university degree. Other demographic information is shown in Table 1.

*Parents’ knowledge of antibiotics*

Most parents often ask their physicians to prescribe antibiotics when their children become ill; 54.4% for the common cold and 47% for both sore throat and cough. When they are nauseous, vomiting, or have diarrhea, 40% of parents ask physicians to order antibiotics. This figure is 4.5% in the case of a runny nose.

**Figure 1:**
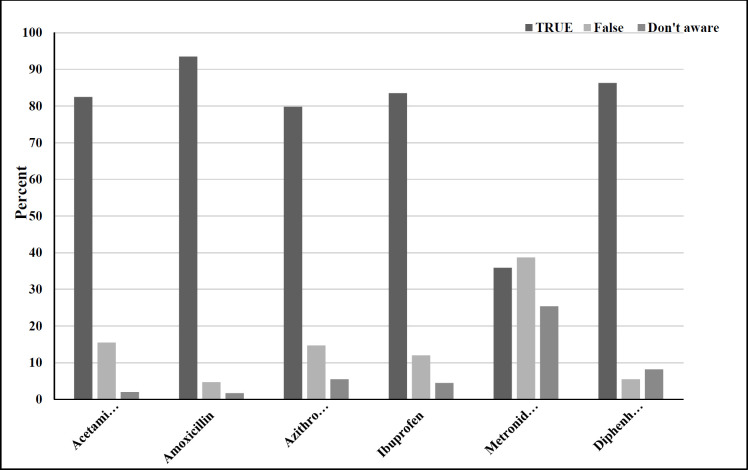
Parents’ ability to distinguish antibiotics from other medicines

On the other hand, 42.5% of participants could properly identify situations where their children needed antibiotics. Parents’ ability to distinguish antibiotics from other types of medicine is shown in Figure 1. All in all, 76.9% of parents properly identified antibiotics. 

The average score of parents’ knowledge of antibiotics regarding administration, indication, storage, and AMR was found to be 9.72 (±SD 2.67) out of a total of 17. Only 31.7% of the parents were aware of the problem of antibiotic resistance, and 19.2% of them did not think that overuse of antibiotics would result in AMR. About 57.2% of the parents had an acceptable knowledge about antibiotics, and 22% and 20.8% had moderate and inadequate knowledge, respectively. Parents’ answers to all questions in part two of the questionnaire are shown in Table 2.

Furthermore, 87.3% of the parents were aware of the importance of administering antibiotics to their children for the whole course of treatment, and, on average, 70.8 were familiar with different storage conditions for antibiotics. Also, 44.25% of the participants had no idea about cautions about the adverse reactions and contraindications of antibiotics written on leaflets. 

Moreover, a significant positive correlation was found between knowledge of antibiotics and some socio-demographic factors such as educational level, occupational status, insurance, and the family’s average monthly expenditure (p<0.05).

*Parents’ practice regarding antibiotics*

In the self-stated parents’ practice questionnaire, 49.9% of the parents answered the questions correctly with the mean score of 3.95 out of a total of 8. 33.9% of parents used antibiotics immediately before the meal if it was mentioned in its order to use before the meal, shown that they had not been educated well about this order. Only 45.6% of the respondents discarded the remainder of antibiotics in case of discontinuation or their physicians prescribing a different antibiotic. Most parents expected the fever to go away automatically after 24-48 hours of starting taking antibiotics, and 74.8% of them referred to doctors again when their children were still feverish. The questions have been presented in Table 3.

Although parents were adequately aware of the adverse reactions of antibiotics, 28.2% of them would not discontinue the medication when an adverse reaction happened. However, 89.3% of them were so sensitive to vomiting immediately after taking antibiotics that they would discontinue the medicine and call their doctors. Moreover, 22.7% would lessen the medication dosage if any adverse reaction happened. 

Regarding the second part of parents’ practice by observation, 68.4% of the participants had evinced a proper practice regarding using antibiotics with a mean score of 5.93 (±1.743) out of a total 10, and 75.5% knew how to prepare the antibiotics powder. However, the majority of parents did not know they had to shake the antibiotics suspension before using it. Also, 63.1% of the respondents referred to leaflet instructions to learn how to keep the antibiotics correctly. The same proportion did not know that 5 milliliters of an antibiotic suspension were equal to a dessertspoon. 

Furthermore, significant correlations were found between parents’ practice regarding the use of antibiotics and their family’s average monthly expenditure, educational level, and occupational status (p < 0.05).

**Table 1:** respondent's demographic characteristic

**Variables**	**Items**	**Frequency N (%)**
Sex	Male	70 (17.5)
	Female	331 (82.5)
Education	High school	145 (36.1)
	Bachelor of Science	213 (53.1)
	Master of Science or above	43 (10.8)
Occupation	Employed	184 (46.2)
	student	47 (11.8)
	jobless	9 (2.3)
	housewife	158 (39.7)
Insurance	Yes	342 (85.9)
	no	53 (13.3)
	Not Available	3 (0.8)
Complementary insurance	Yes	92 (22.9)
	No	302 (75.3)
	Not Available	7 (1.8)
Average family expenditure per month (USD^*^)	Less than 120 USD	33 (8.2)
	120-240 USD	78 (17)
	240-480 USD	180 (44.9)
	More than 480 USD	120 (29.9)

^*^USD: US Dollar.

**Table 2:** Parents’ responses to questions assessing their knowledge of antibiotics

**Not aware (%)**	**False (%)**	**True (%)**	**Questions**
20.9	12.5	66.6	1- Different Antibiotics have the same effect on the treatment of all infections.
32.1	14.5	53.4	2- Antibiotics are effective in both bacteria and viruses.
58.5	15.4	76.1	3-To accelerate treatment, the physician must prescribe antibiotics.
25.2	27.2	47.6	4-Prescribing more than more antibiotics is more effective than prescribing only one antibiotic.
19.4	63.6	17	5-Antibiotics are effective in prevention.
54.1	14.2	31.7	6- I know what antibiotic resistance is.
40.2	19.2	40.6	7- My child’s overuse of antibiotics can cause antibiotic resistance in him/her.
11	21.4	67.6	8- In case the same symptoms as those of a previous disease happen, we should administer the same antibiotics.
46.9	7	46.1	9- Cautions and contraindications mentioned in the drug leaflet are the same.
41.6	6	52.4	10- All adverse reactions mentioned in the drug leaflet happen to everyone.
3.5	9.2	87.3	11- We should continue taking antibiotics to the end of the treatment period.
2.8	67.3	29.9	12- All antibiotics (liquid forms) must be stored in a fridge.
2.8	3.7	93.5	13- Antibiotics must be protected from direct sunlight.
2.8	8.2	89	14- Antibiotics must be stored in cool and dry places.
8.2	55.4	36.4	15- We can take all antibiotics with milk or fruit juice.
19.5	18.9	61.6	16- Reduction in antibiotic usage intervals and increase in prescribed dosage accelerate recovery.
15.2	9.7	75.1	17- To make up for a missed dosage, one should double the next one.
20.8	22	57.2	Total percentage

**Table 3:** Parents’ self-stated practice regarding antibiotics

**Answers** **The correct answers are in parentheses.**	**Questions**
(Yes) NO Sometimes	1- If the antibiotic tastes bad or does not look good, I would not take it.
Yes (NO) Sometimes	2- If the order of an antibiotic is before a meal, I will take it immediately before the meal.
(Fever, diarrhea, acne) Headache, vertigo I don't know.	3- Which of these symptoms could be an adverse effect of an antibiotics?
(Yes) NO I don't know	4- In case of an adverse drug reaction, I would discontinue the antibiotic and call the physician.
(I'd give the prescribed dosage again.) I'd discontinue the antibiotic, and call the physician. I'd give another dosage in the next interval. I don't know.	5- If the child immediately vomits after taking an antibiotic, what would you do?
I'd continue the antibiotic dosage. I'd go to the physician. (I'd give the child acetaminophen to.) I don't know.	6- If your child is taking an antibiotic, and he/she is still feverish after 24-48 hours, what would you do?
Yes (NO) I don't know	7- To minimize the adverse drug reaction, we must lessen drug dosage.
(I’d throw them away) I’d discontinue administering it but keep the remainder for future use. I’d start administering the new type after I am done with the first one. I’d administer both at once.	8- If your physician asks you to stop an antibiotic type or prescribes a different type, what would you do with the remainder of the antibiotics?

## Discussion

Antibiotics are among the most critical medications worldwide due to their great value to public health; however, their efficacy is largely undermined by their irrational use. How patients use antibiotics, and their knowledge and practice regarding their use can go a long way toward their effectiveness in treating some infectious diseases. A positive correlation between patients’ knowledge of antibiotics and practice has been found in previous studies (11). In other words, a good knowledge of the use of antibiotics results in rational use and vice versa. Injudicious use of antibiotics can endanger the patient’s health and cause bacterial resistance. Bacterial resistance to antibiotics has already been reported at alarming levels throughout the world, which is a major health problem (16). With this in mind, this study set out to measure the ill children’s parents’ knowledge and practice regarding antibiotics in Tehran. Moreover, determining the level of parental knowledge of antibiotics and children’s health issues can help identify and meet parents’ training needs. Parents’ low health knowledge can adversely affect their children’s diagnosis and treatment (17). We did the survey in Tehran because it is Iran’s most extensive and most populous city with a very diverse demographic makeup.

The findings of this study showed that most parents would ask their physicians to prescribe antibiotics for their children when they have cold or sore throat. Overall, 31.7% of the parents were familiar with the concept of AMR. However, some of them would administer the same antibiotic to their child as the one they had used before if symptoms were similar to the previous disease. Gualano conducted a meta-analysis of 24 observational studies from January 2000 to November 2013 in Italy. It showed that about 59.4% of the population had some idea about AMR, and 26.9% had no idea that misuse of antibiotics could cause AMR (18).

A small percentage of parents in our study claimed that they would ask physicians to prescribe an antibiotics for accelerating their children’s treatment. However, Miao Yu *et al.* have found that in China about half of the parents had requested an antibiotics prescription (19). Furthermore, Unlike our finding that only 14.5% of the parents considered antibiotics effective in viral infections, in Miao Yu’s study, 79% (19) of the parents considered antibiotics effective in viral infections. This figure was 72.7% in Shanghai’s study (20). In developing countries, self-treatment with antibiotics, especially in viral infections, is common (16). However, an Italian study in 2016 showed that 33% of the participants considered an antibiotic effective in viral infections (8). Therefore, investing in parental education can pay off in the sense that they can easily distinguish viral and bacterial diseases.

Parents’ assumption that the prescription of two or more antibiotics is more effective in the treatment of infectious diseases than the prescription of only one antibiotic was highly consistent with Shanghai’s findings (19). In addition, in our study, most parents believed that antibiotics were effective in prevention, while more than half of the Chinese parents had such a belief (20).

Storage of leftover antibiotics at home is not only a main cause of self-treatment, but it also increases the chance of antibiotics reuse by parents for their children’s treatment, threatening personal and social health because of AMR (21). A global survey has shown that in countries where antibiotics are not supplied with the exact number of pills needed and in packages containing a fixed number, the remaining antibiotics will be kept and reused (21). Stopping treatment earlier than the end of the treatment course is another reason for leftover antibiotics (20). WHO has pointed out that we should take our antibiotics until the end of our treatment period and not discontinue our prescribed antibiotics even when we feel better (4). In Yu *et al.*’s study, two-fifths of the parents believed that antibiotics should be discontinued as soon as symptoms disappeared; this can significantly increase the risk of recurrence and the development of resistant diseases (20). In this study, 9.2% of the parents did not believe that antibiotics must be continued until the end of the treatment course. Moreover, if the prescribed antibiotic was discontinued or replaced by another one, near half of the parents discontinued the antibiotic and kept the remainder for reuse. Revising and modifying leaflets and packaging of antibiotics by the Food and Drug Administration and pharmaceutical companies seem to be a helpful measure to improve patients’ knowledge about keeping and reusing antibiotics.

## Conclusion

This study demonstrates that parents have acceptable practice and knowledge regarding antibiotic consumption. However, there is still room for improvement in upgrading parents’ health performance and increasing their knowledge of some health issues related to antibiotics. Moreover, some visible gaps between the knowledge and practice of parents highlight the necessity of proper translation of knowledge into practice regarding antibiotics for policymakers. 

To ensure successful educational interv-entions, it is necessary to identify the factors contributing to the misuse of antibiotics and the most effective educational programs that best suit every community, which can also be an excellent topic for future research and further exploration. 
